# Safety and economic analysis of the EasyEndo disposable endoscopic cutting and stapling device for VATS lobectomy or segmentectomy in lung cancer patients: a retrospective study

**DOI:** 10.3389/fonc.2023.1247450

**Published:** 2023-08-31

**Authors:** Ziyang Han, Jieming Lu, Shuchen Chen, Shaobin Yu, Peipei Zhang, Mingqiang Kang

**Affiliations:** ^1^ Department of Thoracic Surgery, Fujian Medical University Union Hospital, Fuzhou, China; ^2^ Key Laboratory of Cardio-Thoracic Surgery (Fujian Medical University), Fuzhou, China; ^3^ Key Laboratory of Gastrointestinal Cancer (Fujian Medical University), Ministry of Education, Fuzhou, China; ^4^ Fujian Key Laboratory of Tumor Microbiology, Department of Medical Microbiology, Fujian Medical University, Fuzhou, China

**Keywords:** lung cancer, video-assisted thoracoscopic surgery, EasyEndo, stapler, safety, economic analysis

## Abstract

**Objective:**

The aim of this retrospective study was to investigate the safety and economic aspects of using the EasyEndo disposable endoscopic cutting and stapling device for video-assisted thoracoscopic surgery (VATS) lobectomy or segmentectomy in patients with lung cancer. The choice between the two staplers was influenced by changes in our hospital’s procurement policy; Johnson EC45A was used before January 2022 and was then replaced by the EasyEndo stapler.

**Methods:**

We reviewed and analyzed consecutive patients with lung cancer who underwent VATS segmentectomy from March 2021 to December 2022. Inclusion criteria included patients with suspected non-small cell lung cancer (NSCLC) who were eligible for surgical resection. The surgical procedures were performed using either the EasyEndo or Johnson EC45A staplers. Intraoperative variables, postoperative outcomes, and cost analysis were compared between the two groups.

**Results:**

A total of 1556 patients were included in the study, with 775 patients in the Control group and 781 patients in the EasyEndo group. There were no significant differences in patient characteristics between the two groups. Intraoperative variables, including blood loss, blood transfusion, and operation time, showed no significant differences between the groups. Postoperative outcomes, such as hospital stay, drainage tube placement time, and incidence of complications, were also comparable between the two groups. However, there was a significant difference in the cost of stapler usage, with the EasyEndo group showing a lower cost compared to the Control group.

**Conclusion:**

The EasyEndo disposable endoscopic cutting and stapling device demonstrated comparable safety and effectiveness to the Johnson EC45A stapler in VATS segmentectomy for lung cancer patients. Moreover, the use of the EasyEndo stapler resulted in cost savings, indicating its potential economic benefits for healthcare institutions.

## Introduction

With its daunting mortality rate, lung cancer continues to hold the dubious distinction of being the top contributor to cancer-related fatalities, extinguishing around 350 lives on a daily basis (1). Surgical excision remains the primary treatment modality for early-stage lung cancer (2). In recent years, video-assisted thoracoscopic surgery (VATS) with segmental resection has gained popularity due to the increasing number of patients diagnosed at an early stage, which allows for better preservation of lung function (LF) (3). Moreover, economic analyses have demonstrated that VATS offers lower post-discharge and in-hospital costs compared to open thoracotomy lung resections (4). Therefore, efforts to enhance outcomes while reducing healthcare expenses should focus on targeted interventions that minimize specific complications and maximize cost reduction in areas with the greatest impact (5). Automatic suture devices are now commonly used in procedures involving the excision of lungs and arteries (6). Among these devices, surgical staplers have been widely used for bronchial stump closure, as well as for the resection of pulmonary vessels and tissues in pulmonary surgery (7). These staplers have proven effective in reducing intraoperative bleeding, minimizing pulmonary air leaks, and leading to fewer postoperative complications (6–8).

The EasyEndo disposable endoscopic cutting and stapling device, introduced to the Chinese market in 2020, represents a development based on the company’s earlier generation products. It is primarily designed for resection, transection, and anastomosis of lung, stomach, and intestinal tissues through open or laparoscopic approaches.

The objective of this study is to retrospectively analyze the safety and economic aspects associated with the utilization of the EasyEndo stapling device. By providing valuable insights, this research aims to contribute to the field and advance the development of surgical techniques.

## Materials and methods

### Patients

From March 2021 to December 2022, we retrospectively reviewed and analyzed consecutive patients with lung cancer underwent VATS lobectomy or segmentectomy. All the patients in our study had the stapler with the brand name of EasyEndo or Johnson EC45A during the operation. The choice between two stapler brands, EasyEndo or Johnson EC45A, used during the operation was influenced by changes in our hospital’s procurement policy; Johnson EC45A was used before January 2022 and was then replaced by the EasyEndo stapler.

Inclusion criteria were as follows: (1) patients with imaging findings of lung nodule(s) or mass(es) treated by surgical resection for suspected NSCLC; (2) Preoperative evaluation indicated no unilateral or bilateral lung, mediastinal lymph node or distant metastasis; (3) patients were aged 18 years old or above; (4) no preoperative chemotherapy or radiotherapy, targeted drug therapy, or immunotherapy prior to surgery; (5) no complicated primary malignant tumor in other sites; (6) preoperative ECG, heart color ultrasound, lung function and other examinations indicate good cardiopulmonary function and can tolerate surgical treatment. Blood tests (including routine blood, coagulation function, liver and kidney function, eight surveys before blood transfusion, blood group identification + antibody screening, CEA) and routine stool (routine stool +OB test, routine urine) showed no abnormality and (7) patients and their family members fully understood the surgical plan, were willing to participate in this study, and provided written informed consent. And exclusion criteria were as follows: (1) chest CT scan + enhancement indicated the tumor, adhesion with surrounding tissues, nerves, organs and large vessels, unclear boundary, or multiple metastasis in bilateral lungs and other sites; (2) patients with heart, lung and other important organ dysfunction, and patients who could not tolerate surgery were considered in anesthesia evaluation; (3) patients with previous history of thoracic operation, or patients undergoing open-chest cardiac surgery; (4) intraoperative exploration revealed total thoracic adhesion; (5) intraoperative endoscopic exploration estimated that the tumor could not be completely resected and turned to thoracotomy, or the pleural pericardium and other extensive metastatic patients only underwent thoracoscopic biopsy; (6) a history of other malignant tumors, and the possibility of lung metastasis was considered and (7) patients with serious mental illness.

All the operations were performed by the same surgical team at Union Hospital Affiliated to Fujian Medical University. The patients who had the stapler with the brand name of EasyEndo during operation were assigned to the group of EasyEndo, while the patients who had the stapler with the brand name of Johnson EC45A during operation were assigned to the group of Control. The operative time, intraoperative blood loss, postoperative complications, postoperative hospitalization days, postoperative thoracic drainage volume, days of intrathoracic drainage tube retention were observed and compared between the two groups. The study was approved by institutional ethics board of Fujian Medical University [No.: 2023KY112] and informed consent was taken from all the patients.

### Surgical technique

After general anesthesia, the patient was placed a left or right lateral position at 90°. The surgeon was located on the ventral side of the patient, one assistant was located on the back of the patient, and the other assistant was located behind the surgeon. (1) Selection of incision: firstly, a 2cm transverse incision as a probe hole was made at the midaxillary line and the 7th intercostal junction. 2-3cm operating holes were made at the anterior axillary line and the 4th intercostal junction, and at the 9th intercostal junction of the posterior axillary line. (2) For VATS lobectomy, the procedure followed the conventional approach with a 3-hole technique. The vessels and bronchus to the lobe were divided with a stapler device. The lobar fissures were also divided with a stapler, and the lobe was removed. The chest was then drained, and the incisions were closed. (3) For segmentectomy, the location of lung nodules was determined. The corresponding pulmonary segmental arteries, veins, and bronchus were exposed. The corresponding pulmonary segmental arteries, veins, and bronchus were severed. The “dilatation-collapse” method was used to determine the intersegmental plane, and the corresponding lung segments were severed with staplers (6). Finally, adequate hemostasis and chest closure were performed after the corresponding lung tissue was removed.

### Endoscopic linear staplers

The control group utilized the Johnson EC45A linear stapler, while the EasyEndo group used the U12M60 stapler.

**Figure 1:**
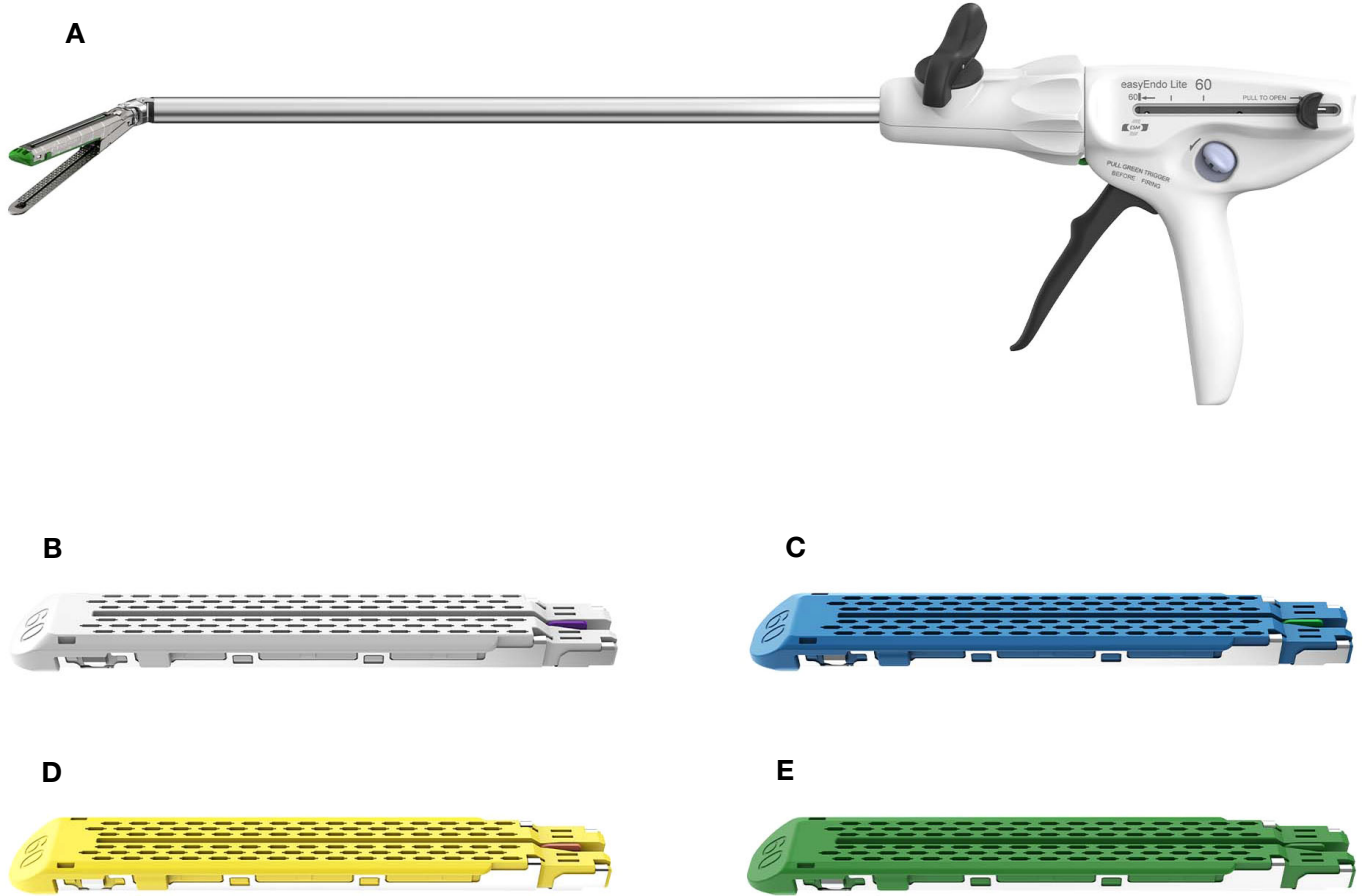
EasyEndo disposable endoscopic stapler and its components. **(A)** Disposable endoscopic stapler; **(B–E)** the components (Model: N60W, N60B, N60C, N60G).

During the surgery, EasyEndo disposable endoscopic cutting and stapling device (Model: U12M60) (**Figure 1A**) and its components (Model: N60W, N60B, N60C, N60G) (**Figures 1B–E**) (Shanghai Eseemed Medical Technology Co., Ltd., Shanghai, China; registration number: 20202020188) were used in the EasyEndo group for the cutting and closure of lung tissue, bronchi, pleura, and other areas.

### Measures and outcome variables

The primary measures included intraoperative blood loss and intraoperative blood transfusion, both indicative of the safety and efficacy of the stapler used. The secondary measures were postoperative hospital stay and postoperative drainage tube placement time, which are crucial indicators of postoperative recovery and potential complications. Criteria for patient discharge and chest tube removal were as follows: patients were discharged when they were afebrile, could mobilize independently, had controlled pain, and had no evidence of infection or major complications. The chest tube was removed when the volume of pleural drainage was less than 200 mL/day, there was no air leak, and the lung was fully re-expanded. We also closely monitored the incidence of postoperative complications, such as bronchial fistula, pleural effusion, and pulmonary infection, which are common adverse events following thoracic surgery and significantly impact patient prognosis. Readmissions within 30 days post-discharge were also recorded as a proxy for postoperative complications not identified during the initial hospital stay. The cost of the use of staplers was calculated and compared between the two groups to assess the economic efficiency of the two staplers.

These measures were selected because they provide a comprehensive assessment of the safety, efficacy, and cost-effectiveness of the two stapler devices used in VATS lobectomy or segmentectomy. By comparing these variables between the two groups, we aimed to provide valuable insights into the optimal choice of stapler for thoracic surgery.

### Data collection and statistical analysis

Continuous variables were presented as mean value ± standard deviation, while categorical variables were reported as percentages. The statistical significance of differences between continuous variables was evaluated using Student’s t-test or Mann-Whitney U-test, whereas categorical variables were analyzed using Pearson’s χ2 test or Fisher’s exact test. All tests were two-sided and a p-value of less than 0.05 was considered statistically significant. Data analysis was performed using R Statistics software version 4.2.1.

## Results

### Clinical characteristics of patients

The clinical characteristics of the patient are shown in **Table 1**. The patient characteristics, including age, BMI, gender, tobacco use, tumor stage, pathology, and preoperative complications, were analyzed and compared between the Control and EasyEndo groups. There were no significant differences observed in any of these variables between the two groups (all p > 0.05). In the Control group, there were 2 patients who originally had brain metastasis, while in the EasyEndo group, there was 1 patient. After preoperative neoadjuvant treatment, their brain lesions disappeared, making them eligible for surgery. The mean age of the total sample was 56.1 years, with similar values in the Control (56.1 years) and EasyEndo (56.2 years) groups (p = 0.895). The mean BMI was 23.0 kg/m^2 in the total sample, with no significant difference between the groups (Control: 22.9 kg/m^2, EasyEndo: 23.1 kg/m^2, p = 0.276). The gender distribution was similar between the Control and EasyEndo groups, with approximately 58% of patients being female (p = 0.98). Additionally, there were no significant differences in tobacco use, tumor stage, pathology, or the presence of comorbidity between the two groups (all p > 0.05).

**Table 1:** Perioperative clinical characteristics of the patients.

Variable	Total	Control	EasyEndo	P.value
	(N=1556)	(N=775)	(N=781)	
Age (years)				
Mean (SD)	56.1 (11.8)	56.1 (11.9)	56.2 (11.6)	0.895
BMI (kg/m^2)				
Mean (SD)	23.0 (3.58)	22.9 (4.05)	23.1 (3.04)	0.276
Gender				
Female	906 (58.2%)	452 (58.3%)	454 (58.1%)	0.98
Male	650 (41.8%)	323 (41.7%)	327 (41.9%)	
Tabacco				
No	1198 (77.0%)	599 (77.3%)	599 (76.7%)	0.827
Yes	358 (23.0%)	176 (22.7%)	182 (23.3%)	
Tumor.stage				
Stage I	841 (54.0%)	423 (54.6%)	418 (53.5%)	0.713
Stage II~IV	715 (46.0%)	352 (45.4%)	363 (46.5%)	
Pathology				
Adenocarcinoma	937 (60.2%)	462 (59.6%)	475 (60.8%)	0.664
Non-adenocarcinoma	619 (39.8%)	313 (40.4%)	306 (39.2%)	
Comorbidity				
Coronary heart disease	110 (7.1%)	53 (6.8%)	57 (7.3%)	0.935
Diabetes	169 (10.9%)	85 (11.0%)	84 (10.8%)	
Hypertension	424 (27.2%)	209 (27.0%)	215 (27.5%)	
Others	47 (3.0%)	21 (2.7%)	26 (3.3%)	
None	806 (51.8%)	407 (52.5%)	399 (51.1%)	
Surgical.Type				
Left-side Segmentectomy	215 (13.8%)	105 (13.5%)	110 (14.1%)	0.998
Right-side Segmentectomy	245 (15.7%)	124 (16.0%)	121 (15.5%)	
Right Upper Lobectomy	309 (19.9%)	155 (20.0%)	154 (19.7%)	
Right Middle Lobectomy	54 (3.5%)	28 (3.6%)	26 (3.3%)	
Right Lower Lobectomy	370 (23.8%)	186 (24.0%)	184 (23.6%)	
Left Upper Lobectomy	225 (14.5%)	111 (14.3%)	114 (14.6%)	
Left Lower Lobectomy	138 (8.9%)	66 (8.5%)	72 (9.2%)	

BMI, body mass index.

These results indicate that the patient characteristics were well-balanced between the Control and EasyEndo groups, minimizing potential confounding factors and allowing for a more accurate comparison of surgical outcomes.

### Intra/Post-operative outcomes

#### Intraoperative variables

**Table 2** shows the intra/post-operative outcomes. The comparison of intraoperative variables between the Control and EasyEndo groups showed no significant differences. The mean intraoperative blood loss was 37.5 ml in the total sample, with no significant difference observed between the two groups (Control: 37.0 ml, EasyEndo: 37.9 ml, p = 0.645). Intraoperative blood transfusion was required in only one case (0.1%) in the EasyEndo group, while no transfusions were needed in the Control group (p = 1). The operation time was similar between the two groups, with a mean of 106 minutes (Control: 106 minutes, EasyEndo: 106 minutes, p = 0.988).

**Table 2:** A comparison of intra- and postoperative outcomes.

Variable	Total	Control	EasyEndo	P.value
	(N=1556)	(N=775)	(N=781)	
Intraoperative.blood.loss (ml)				
Mean (SD)	37.5 (40.4)	37.0 (33.2)	37.9 (46.5)	0.645
Intraoperative.blood.transfusion				
No	1555 (99.9%)	775 (100%)	780 (99.9%)	1
Yes	1 (0.1%)	0 (0%)	1 (0.1%)	
Operation.time (minutes)				
Mean (SD)	106 (52.7)	106 (53.0)	106 (52.4)	0.988
Postoperative.hospital.stay (days)				
Mean (SD)	4.58 (2.70)	4.56 (2.72)	4.60 (2.68)	0.796
Postoperative.drainage.tube.placement.time (days)				
Mean (SD)	3.22 (1.98)	3.23 (2.04)	3.22 (1.92)	0.905
Postoperative. bronchial fistula				
No	1556 (100%)	775 (100%)	781 (100%)	0.879
Postoperative.pleural.effusion				
No	1534 (98.6%)	765 (98.7%)	769 (98.5%)	0.844
Yes	22 (1.4%)	10 (1.3%)	12 (1.5%)	
Postoperative.pulmonary.infection				
No	1539 (98.9%)	767 (99.0%)	772 (98.8%)	1
Readmissions				
No	1517 (97.5%)	757 (97.7%)	760 (97.3%)	0.764
Yes	39 (2.5%)	18 (2.3%)	21 (2.7%)	
Cost of the use of staplers (USD)				
Mean (SD)	1993.72 (698.50)	2356.22 (681.77)	1631.23 (503.31)	<0.001*

*; P<0.05.

#### Postoperative outcomes

The postoperative variables showed no significant differences between the Control and EasyEndo groups. The mean postoperative hospital stay was 4.58 days in the total sample, with no significant difference between the groups (Control: 4.56 days, EasyEndo: 4.60 days, p = 0.796). Similarly, there were no significant differences in the mean postoperative drainage tube placement time between the two groups (Control: 3.23 days, EasyEndo: 3.22 days, p = 0.905). The incidence of postoperative complications, including bronchial fistula, pleural effusion, pulmonary infection, and readmissions, did not differ significantly between the two groups.

#### Cost analysis

The cost analysis revealed a significant difference in the cost of stapler usage between the Control and EasyEndo groups. The mean cost of the use of staplers in the total sample was 1993.72 USD, with the EasyEndo group showing a significantly lower cost compared to the Control group (Control: 2356.22 USD, EasyEndo: 1631.23 USD, p < 0.001). The cost reduction associated with the use of EasyEndo staplers suggests substantial savings potential for healthcare institutions and the healthcare system.

## Discussion

In our study, we made an observation that the total cost associated with the use of staplers in the EasyEndo group was significantly lower compared to the Control group. However, our results did not demonstrate any statistically significant differences in various factors including operation time, intraoperative blood loss, postoperative hospital stays, postoperative drainage tube placement time, intraoperative blood transfusion, and intraoperative bronchial fistula.

Since the concept for the surgical stapler was first developed in Hungary in 1908 by Hültl, a professor and surgeon (9), linear staplers have become a common automatic suture tool in thoracoscopic surgical systems now (10). Air leakage is a common postoperative complication that can increase the risk of serious complications, such as postoperative pneumothorax (11). Previous studies have shown that the use of staplers dramatically decreased postoperative air leakage rate and the operation time (12–14). Our review and analysis of previous research data (15) also indicated satisfactory results in terms of operation time and postoperative air leakage rates for both groups. Additionally, we utilized human fibrinogen and thrombin during the operations in both groups, which has been shown to reduce postoperative air leakage rates in previous studies (16).

Because of the restrictions on the moving forceps and operative vision, the management of the vascular injury during thoracoscopic surgery is particularly important compared with that during a thoracotomy. In particular, bleeding from the pulmonary arteries (PAs), which frequently results in a situation in which life is in danger, has raised questions about the safety of VATS (17). In our study, there was no significant difference in intraoperative blood loss between the EasyEndo group and the Control group. Although one patient required intraoperative blood transfusion due to a massive hemorrhage during surgery, the bleeding was caused by a blood vessel mutation during lymph node dissection and not by the use of a stapler. Therefore, we believe that the use of the EasyEndo linear stapler in VATS can provide a comparable level of safety to the Control linear stapler.

Enhanced recovery after surgery (ERAS) has become widely adopted and aims to reduce surgical stress responses, complications, medical costs, and promote patient recovery through optimized perioperative management measures (18). The removal of drainage tubes is mainly dependent on the presence of persistent air leakage after surgery, which is a major cause of prolonged postoperative hospital stays (19). In our study, the average postoperative hospital stays in both groups were 4.6 and 4.5 days, respectively, with no significant difference. The duration of postoperative hospital stay is closely related to the drainage tube retention time. Our study showed no significant difference in the incidence of postoperative complications and readmissions between the two groups. The incidence of postoperative complications and readmissions aligns with previous studies on ERAS (20). Therefore, our patients using the EasyEndo or Control linear stapler in this study were able to achieve ERAS effectively.

The use of EasyEndo staplers was associated with a 15.4% lower cost compared to Echelon staplers, indicating significant potential for cost savings in healthcare institutions and systems.

Our findings revealed a significant reduction in the total cost of stapler usage in the EasyEndo group. This reduction in cost could potentially result in substantial savings for both healthcare providers and patients. Several factors may contribute to the decreased cost, including the availability of more affordable staplers, lower rates of complications necessitating additional interventions, or more efficient resource utilization in the EasyEndo group.

These cost savings have important implications for healthcare systems, as they can lead to more efficient allocation of resources and improved financial sustainability. By utilizing EasyEndo staplers, healthcare providers may be able to optimize their budgetary allocations and allocate the saved funds towards other essential healthcare needs. Additionally, patients can benefit from reduced financial burdens associated with surgical procedures, making healthcare more accessible and affordable.

It is important to consider the potential long-term economic benefits associated with the use of EasyEndo staplers. Further studies and cost-effectiveness analyses are warranted to comprehensively evaluate the economic impact and sustainability of incorporating EasyEndo staplers into routine clinical practice. These analyses can provide valuable insights for healthcare decision-makers and policy developers in optimizing surgical interventions and maximizing cost-efficiency.

The limitations of our study should be acknowledged. Firstly, it is important to note that our research was conducted at a single institution, which may limit the generalizability of our findings to other healthcare settings. Secondly, the sample size in our study was relatively small. Although we observed significant differences between EasyEndo and Control groups, the limited sample size increases the possibility of type II errors and restricts the statistical power of our conclusions. Furthermore, it is worth mentioning that our study relied on subjective assessments for certain outcomes, such as intraoperative bleeding and operation time. The lack of a standardized approach for measuring these parameters in real-world hospital settings may have introduced variability and potential measurement biases. Lastly, the follow-up period in our study was relatively short, and the long-term effects and durability of the stapler were not assessed. Therefore, future studies with extended follow-up durations are necessary to provide a more comprehensive understanding of the long-term outcomes associated with the use of the stapler.

In conclusion, our study provides compelling evidence supporting the use of EasyEndo staplers in VATS procedures, as they demonstrate comparable safety profiles to Johnson EC45A staplers, while also offering a more cost-effective alternative. By confirming the efficacy and affordability of EasyEndo staplers, our findings have significant implications for surgical practice and healthcare systems. This study contributes to the growing body of literature supporting the use of EasyEndo staplers as a viable option in thoracic surgery, highlighting their potential to enhance patient outcomes while optimizing resource utilization. Further research and long-term follow-up studies are warranted to validate and expand upon our findings, ultimately guiding clinical decision-making and advancing surgical techniques.

## Data availability statement

The raw data supporting the conclusions of this article will be made available by the authors, without undue reservation.

## Author contributions

Contributions: (I) Conception and design: MK and ZH. (II) Administrative support: ZH. (III) Provision of study materials or patients: JL and SC. (IV) Collection and assembly of data: JL, PZ, and SY. (V) Data analysis and interpretation: SY. (VI) Manuscript writing: all authors. (VII) Final approval of manuscript: all authors.
